# Penetration into Cancer Cells via Clathrin-Dependent Mechanism Allows L-Asparaginase from *Rhodospirillum rubrum* to Inhibit Telomerase

**DOI:** 10.3390/ph13100286

**Published:** 2020-09-30

**Authors:** Anna A. Plyasova, Marina V. Pokrovskaya, Olga M. Lisitsyna, Vadim S. Pokrovsky, Svetlana S. Alexandrova, Abdullah Hilal, Nikolay N. Sokolov, Dmitry D. Zhdanov

**Affiliations:** 1 Institute of Biomedical Chemistry, Pogodinskaya st. 10/8, 119121 Moscow, Russia; annaplyasova13@gmail.com (A.A.P.); ivan1190@ya.ru (M.V.P.); mbt12@yandex.ru (S.S.A.); abdulla.hilal@inbox.ru (A.H.); nikolai.sokolov@ibmc.msk.ru (N.N.S.); 2 International Biotechnology Center “Generium” LLC, Vladimirskaya st. 14, 601125 Volginsky, Russia; lisitsynaom@gmail.com; 3 N.N. Blokhin Cancer Research Center, Kashirskoe Shosse 24, 115478 Moscow, Russia; v.pokrovsky@ronc.ru; 4 Department of Biochemistry, Рeoples Friendship University of Russia (RUDN University), Miklukho-Maklaya st. 6, 117198 Moscow, Russia

**Keywords:** *Rhodospirillum rubrum*, L-asparaginase, telomerase, endocytosis, clathrin, dynamin

## Abstract

The anticancer effect of L-asparaginases (L-ASNases) is attributable to their ability to hydrolyze L-asparagine in the bloodstream and cancer cell microenvironment. *Rhodospirillum rubrum* (RrA) has dual mechanism of action and plays a role in the suppression of telomerase activity. The aim of this work was to investigate the possible mechanism of RrA penetration into human cancer cells. Labeling of widely used L-ASNases by fluorescein isothiocyanate followed by flow cytometry and fluorescent microscopy demonstrated that only RrA can interact with cell membranes. The screening of inhibitors of receptor-mediated endocytosis demonstrated the involvement of clathrin receptors in RrA penetration into cells. Confocal microscopy confirmed the cytoplasmic and nuclear localization of RrA in human breast cancer SKBR3 cells. Two predicted nuclear localization motifs allow RrA to penetrate into the cell nucleus and inhibit telomerase. Chromatin relaxation promoted by different agents can increase the ability of RrA to suppress the expression of telomerase main catalytic subunit. Our study demonstrated for the first time the ability of RrA to penetrate into human cancer cells and the involvement of clathrin receptors in this process.

## 1. Introduction

Normal and tumor cells require the amino acid L-asparagine for their metabolic needs. Normal cells can synthesize L-asparagine for their growth through the enzyme asparagine synthetase. Neoplastic cells lack the ability to synthesize asparagine due to the absence or shortage of L-asparagine synthetase and are dependent on an exogenous supply of this amino acid from the bloodstream [1].

L-asparaginase (L-ASNase), also known as L-asparagine amidohydrolase (EC 3.5.1.1), is the first therapeutic enzyme with antineoplastic properties. A common therapeutic role of L-ASNase is based on its ability to hydrolyze L-asparagine to L-aspartate and ammonia. The exposure of tumor cells, mainly leukemic cells, to L-ASNase leads to cancer cell starvation resulting in their death [2].

L-ASNases have been identified in mammals, birds, plants, fungi, and a wide range of bacteria [3,4]. To date, dozens of microbial sources of L-ASNases have been revealed, though not all of them demonstrated cytotoxicity against leukemic cells or tumor inhibitory effects [4,5].

Besides the well-studied anti-proliferating effects of L-ASNases, which are believed to be caused by asparagine deprivation in the tumor cell environment, several alternative mechanisms have also been investigated. Other L-ASNase substrates include L-glutamine, D-asparagine, succinic acid monoamide and asparaginyl-tRNA [6,7]. Thus, the antiproliferative or side effects may appear due to their degradation. In 1970, it was shown that L-ASNase from *E. coli* may release carbohydrates from α2-HS-glycoprotein fetuin, suggesting that hydrolysis of cell membrane glycoproteins and inhibition of their synthesis by the enzyme can result in cell lysis [8]. This enzyme could also inhibit glycoprotein biosynthesis and lead to membrane sensitivity due to the specific effect on the concanavalin A receptor in the sensitive and resistant L5178Y murine lymphoma cell line [9]. These observations denote the existence of complex mechanisms of action of at least one L-ASNase to a given cell line.

A very surprising cytotoxic asparagine-independent mechanism was described for a *Rhodospirillum rubrum* mutant L-ASNase with E149R, V150P, and F151T amino acid substitutions (RrA). RrA demonstrated regulatory capacity and could suppress telomerase activity in a number of human cancer cell lines, normal activated CD4^+^ T lymphocytes and xenografts of human solid tumors [10,11]. The role of RrA in telomerase suppression indirectly indicates its intracellular or even intranuclear localization as well as its ability to penetrate into the cellular membrane. The mechanism of its penetration into cells remains unclear. In this work we demonstrated the cellular localization of RrA in human cancer cells and the role of clathrin receptors during RrA penetration into cells.

## 2. Results

### 2.1. The Ability of RrA but No Other L-ASNases to Suppress Telomerase Activity

**Figure 1:**
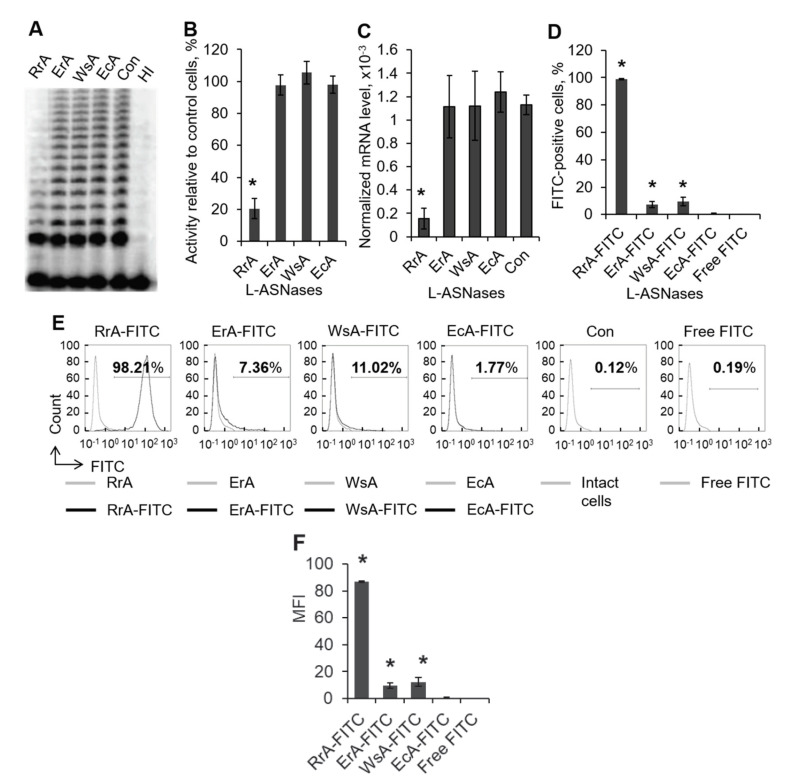
The ability of RrA, but no other L-asparaginases, to suppress telomerase activity. Jurkat cells were incubated with L-ASNases or L-ASNases conjugated with FITC for 12 h. (**A**) Telomerase activity determined by TRAP assay in cells incubated with L-ASNases. (**B**) Results of TRAP quantification by densitometry. (**C**) Levels of hTERT mRNA expression normalized relative to the expression of the reference gene 18S. (**D**) Flow cytometry results for cells incubated with L-ASNases or FITC-conjugated L-ASNases. (**E**) Representative flow cytometry diagrams for incubated cells. (**F**) Mean fluorescence intensity of FITC-positive cells. *n* = 4. * *p* ≤ 0.05 vs. control cells treated with non-conjugated L-ASNase. HI, sample with heat-inactivated telomerase.

It was previously shown that RrA can inhibit telomerase in cancer cells and normal human lymphocytes by inhibiting the expression of its catalytic subunit hTERT (human telomerase reverse transcriptase) [10,11]. We checked whether other L-ASNases have similar effects on telomerase by incubating Jurkat cells with enzymes of different origins. Only RrA was able to inhibit telomerase activity in up to 14.0–26.8% of control cells, while the rate of telomerase activity in cells incubated with ErA, WsA and EcA was not different from control cells (Figure 1A,B). Measurement of hTERT mRNA levels by real-time RT-PCR revealed significant down-regulation of hTERT expression in cells incubated with RrA (Figure 1C). ErA, WsA and EcA showed no ability to suppress hTERT expression.

The potency of RrA to suppress telomerase, which is active in cell nucleus, indirectly indicates its ability to penetrate cell membrane. To investigate the capacity of L-ASNases to interact with cells, we conjugated each enzyme with FITC. The conjugation efficiency (FITC/protein, F/P ratio) is shown in Table 1 and varied in the range of 0.14–0.19, which is an optimal ratio for flow cytometry and fluorescent microscopy [12].

Jurkat cells were incubated with each FITC-conjugated L-ASNase for 12 h and the proportion of FITC-positive cells was measured by flow cytometry. Almost 100% of cells were FITC-positive after incubation with RrA-FITC (Figure 1D,E). A significant increase in FITC-positive cells was also observed in cells treated with FITC-conjugated ErA or WsA, but the rate did not exceed 10%. Incubation of cells with EcA-FITC did not lead to an increase in FITC-positive cells. Mean fluorescence intencity (MFI) was the highest in RrA-FITC-treated cells (88.4 arbitrary units (AU), while the rate of MFI for cells treated with other L-ASNase did not exceed 20 AU (Figure 1F). These results indicate that the ability of RrA to suppress telomerase activity is associated with its capacity to interact with cells.

### 2.2. The Rate of Telomerase Inhibition in Different Cell Lines Corresponds with the Ability of RrA to Interact with Cells

**Figure 2:**
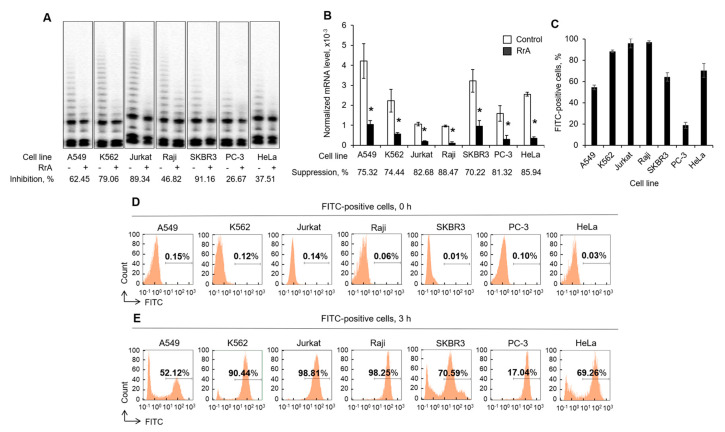
Inhibition of telomerase activity by RrA is related to its ability to interact with cells.Different cell lines were incubated with RrA or FITC-conjugated RrA for 3 h. (**A**) Telomerase activity determined by TRAP assay in cells incubated with RrA. (**B**) HTERT mRNA expression levels measured by real-time RT-PCR and normalized relative to the expression of the reference gene 18S. (**C**) Flow cytometry results for cells incubated with FITC-conjugated RrA. Representative flow cytometry diagrams for (**D**) control cells and (**E**) cells incubated with FITC-conjugated RrA. *n* = 4. * *p* ≤ 0.05 vs. control cells.

We tested the ability of RrA to down-regulate hTERT expression and to inhibit telomerase activity in different cancer cell lines after 3 h of incubation. TRAP assay demonstrated that the most significant inhibition of telomerase activity was in Jurkat and SKBR3 cells, while PC-3 cells showed the lowest rate of inhibition (Figure 2A). The rate of suppression of hTERT expression was not strongly associated with the rate of telomerase inhibition; the most significant suppression was in Raji cells while the lowest inhibition was observed in SKBR3 cells (Figure 2B). However, incubation of cells with FITC-conjugated RrA followed by flow cytometry revealed that RrA had the strongest interaction with Jurkat cells, while PC-3 cells showed the weakest interaction with RrA (Figure 2C–E).

### 2.3. RrA, But Not Other L-ASNases, Is Able to Get Inside Cells

**Figure 3:**
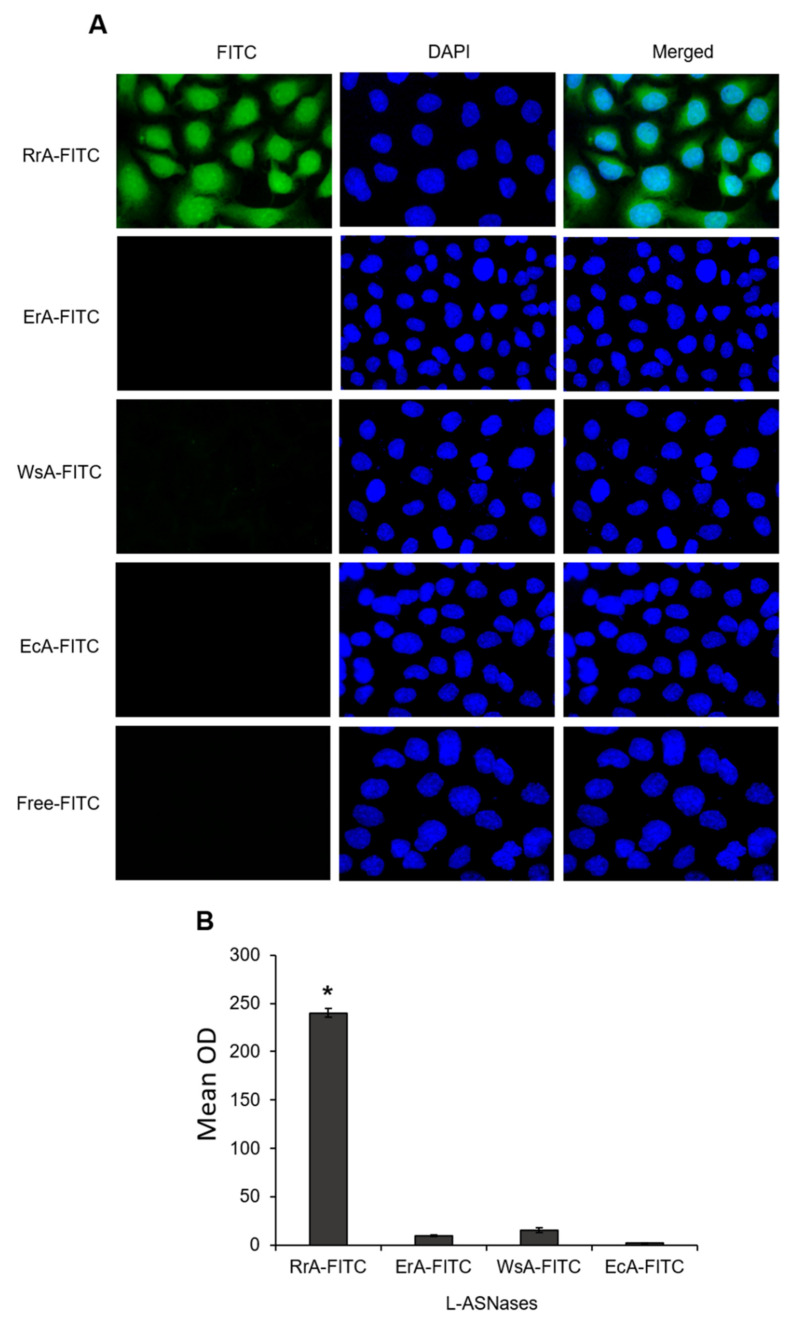
Induction of FITC-positive cells after treatment with FITC-conjugated L-ASNases. (**A**) Representative fluorescent microscopy images of SKBR3 cell incubated with different FITC-conjugated L-ASNases for 12 h and stained with DAPI (green, FITC, and blue, DAPI, magnification ×40). (**B**) Quantification of the FITC mean optical density (OD) in cells. * *p* ≤ 0.05 vs. cells incubated with free FITC.

The suppression of hTERT expression by RrA and its interaction with different cell lines indicates the ability of the enzyme to penetrate through cell membranes. We examined the ability of different FITC-conjugated L-ASNases to translocate into SKBR3 cells. Quantitative fluorescent microscopy demonstrated that FITC-positive cells were significantly induced after 12 h treatment with RrA only (Figure 3). The incubation of cells with other L-ASNases did not lead to induction in FITC-positive cells, indicating their inability to translocate inside the cells. These results are in accordance with the inability of ErA, WsA or EcA to interact with cells, which was demonstrated by flow cytometry (Figure 1D,E).

### 2.4. RrA Protein Mainly Localizes in the Nucleus and Cytoplasm and Has Two Potential Nuclear Localization Signals

**Figure 4:**
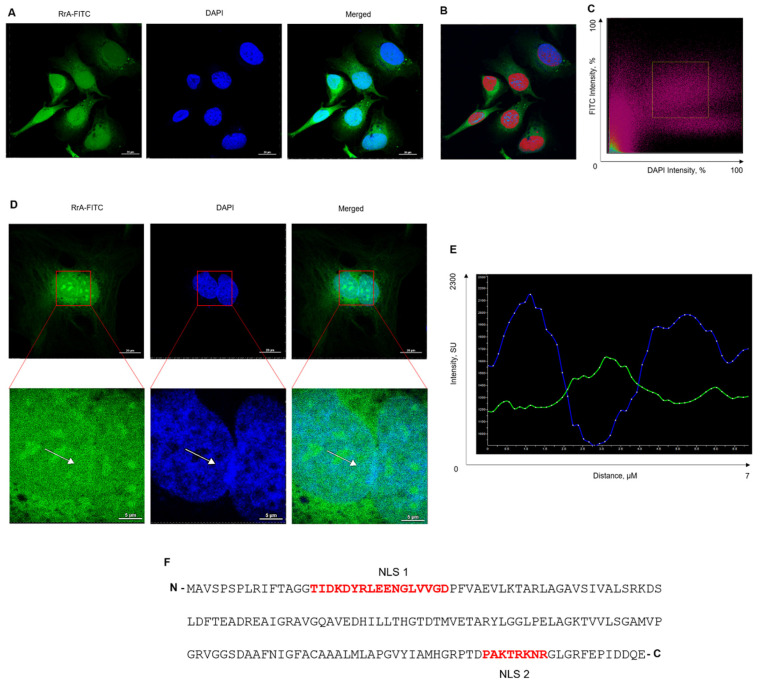
Intracellular localization of RrA-FITC. SKBR3 cells were incubated with RrA-FITC for 12 h and fixed in 4% formalin and DNA was counterstaining with DAPI, followed by confocal scanning fluorescent microscopy. (**A**) Representative confocal microscopy images of incubated cells (maximal projection of *Z*-stack; *green*, RrA-FITC; *blue*, DAPI; magnification of ×60; oil immersion; scale bars are 20 μm). (**B**) Pseudocolored intracellular distribution of co-localized signals from DAPI and FITC (shown in *magenta*) created by co-localization analysis. (**C**) Scattergram presenting the overall relationship between the intensities of signals from DAPI and FITC. The distribution of co-localized signals is shown in *magenta*. (**D**) Single cell confocal images (bars are 5 μm) and magnified regions of interest (bars are 5 μm) showing nucleolar localization of RrA-FITC (maximal projection of *Z*-stack, magnification ×60, oil immersion). (**E**) Fluorescence intensity profile along the white arrow shown in (**D**). The *X*-axis of the profile corresponds to the distance in microns and the Y-axis corresponds to the fluorescence intensity in standard units (SU). Green line, profile of FITC-signal; blue line, profile of DAPI. (**F**) Amino acid sequences of RrA and predicted nuclear localization signals. Predicted nuclear localization signals (NLS 1 and NLS 1) are shown in red bold font. N and C in black bold font denote the N- and C-ends of the protein, respectively.

Confocal scanning microscopy of SKBR3 cells incubated with RrA-FITC mainly showed cytoplasmic and nuclear localization of the FITC-signal (Figure 4A). The potential relationship between RrA-FITC and cell nuclei was assessed by the Pearson’s and Manders’ colocalization algorithms (Figure 4B,C). Pearson’s pixel-to-pixel correlation coefficient between RrA-FITC and DAPI was 0.695. To measure the degree of localization and overlap between RrA-FITC and DAPI, Manders’ correlation was carried out. Manders’ co-localization index for total cellular RrA-FITC versus DAPI was 0.762.

We observed nucleolar localization of RrA-FITC (Figure 4D). In the green channel, an increased intensity of fluorescence was observed in the nucleolus region (or euchromatin). The proof of this is the fluorescence intensity profile along the white arrow (shown in Figure 4D). At the places of decreased intensity in the DAPI channel (nucleolus or euchromatin), the intensity of FITC fluorescence is significantly increased (Figure 4E).

Two possible NLSs have been predicted in the RrA amino acid sequence (Figure 4F). Three NLS prediction servers, PSORT II, NLStradamus and SeqNLS, predicted the same putative NLS in the RrA amino acid sequence, 153-PAKTRKNR-160 (with the highest match score of 0.433, Table 2). Another NLS, 16-TIDKDYRLEENGLVVGDP FVAEVLKTARL-45, was predicted by the cNLS Mapper program. The presence of a putative NoLS was not identified in the RrA amino acid sequence by the NoLS prediction server NOD. Computational analysis revealed neither NLS nor NoLS amino acid sequences in ErA, WsA or EcA. The amino acid sequences of the investigated L-ASNases are shown in Table S1 in the Supplementary file.

### 2.5. Clathrin but Not Caveolin Receptors Mediate RrA Interactions with Cells

**Figure 5:**
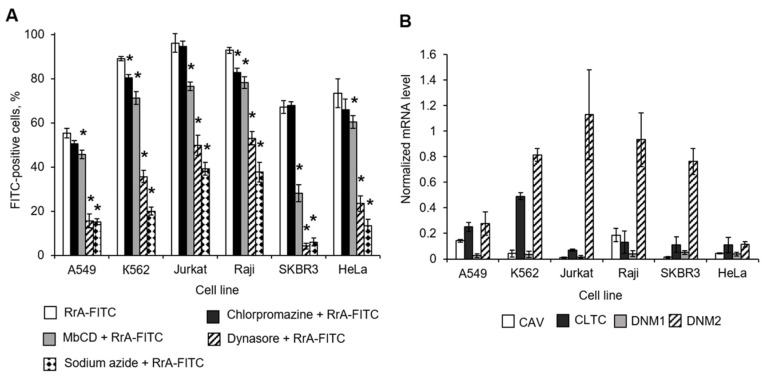
Inhibition of clathrin receptors affects RrA interaction with cells. Different cell lines were exposed to the endocytosis receptor inhibitors for 30 min and incubated with FITC-conjugated RrA for 3 h, followed by flow cytometry. (**A**) Flow cytometry results. (**B**) Expression of CAV, CLTC DNM1 and DNM2 mRNA levels in different cell lines. The expression levels were normalized relative to the expression of the reference gene 18S. *n* = 4. * *p* ≤ 0.05 vs. cells treated with FITC-conjugated RrA.

Receptor-mediated endocytosis involves clathrin-dependent (including dynamin-dependent) and -independent (caveolin-dependent) mechanisms which allow different proteins to pass through the cell membrane [13]. We exposed cells to endocytosis receptor inhibitors and incubated them with FITC-conjugated RrA followed by flow cytometry. The selective clathrin receptor inhibitor chlorpromazine demonstrated a weak ability to inhibit the interaction of RrA with cells (Figure 5A, Table 3). Inhibitor concentrations were chosen from preliminary experiments (Figure S1 in Supplementary file). The non-selective inhibitor of caveolin receptor MbCD showed rather moderate activity. The highest activity to suppress RrA-cell interactions was demonstrated by the selective inhibitor of dynamin-dependent endocytosis Dynasore. The most sensitive cells were SKBR3 and A549 cells. Sodium azide (NaN_3_), known as a non-selective inhibitor of all types of ATP-dependent endocytosis, significantly inhibited the interaction of RrA-FITC with cells and was used as a positive control. Representative flow cytometry diagrams are presented in Figure S2 in the Supplementary file. The inhibitors may be toxic for cells that may result on membrane permeability. Data shown in Figure S3 in the Supplementary file demonstrated that used concentrations of the inhibitors were not toxic for cells. Quantification of clathrin (CLTC), caveolin (CAV) receptor or dynamin protein (DNM1 and DNM2) mRNA levels, which are parts of clathrin receptors, showed a significant predominance of DMN2 mRNAs in cells (Figure 5B). We found no correspondence between CAV, CLTC or DMN1 expression and the ability of RrA-FITC to interact with cells or the inhibition of this process in cells. However, DNM2 expression demonstrated good correspondence with the RrA-FITC interaction efficiency. Jurkat and Raji cells, which had the highest sensitivity to RrA-FITC (Figure 2E), showed the highest DMN2 mRNA levels, while cells with the lowest sensitivity, A549 and HeLa cells, demonstrated the lowest DNM2 mRNA levels.

### 2.6. Telomerase Suppression by RrA Is Abolished by the Inhibition of Clathrin Receptors

**Figure 6:**
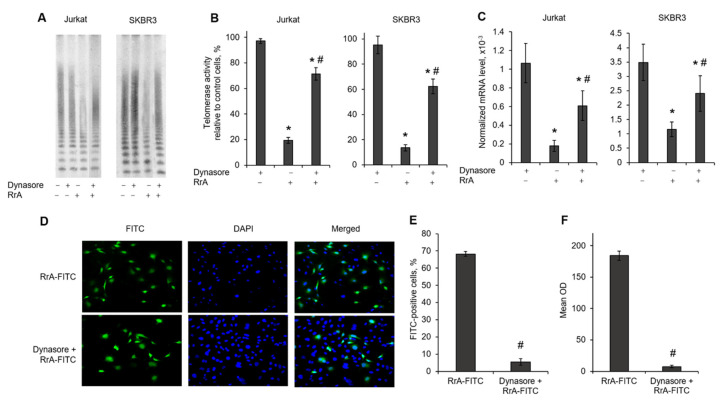
Inhibition of clathrin receptors affects telomerase suppression with RrA. Table 3. cell lines were incubated in the presence of Dynasore and RrA for 3 h, followed by telomerase assay and real-time RT-PCR. (**A**) Telomerase activity determined by TRAP assay in incubated cells. (**B**) Results of TRAP quantification by densitometry. (**C**) HTERT mRNA expression levels normalized relative to the expression of the reference gene 18S. *n* = 4. * *p* ≤ 0.05 vs. cells exposed to Dynasore only. # *p* ≤ 0.05 vs. cells incubated with RrA. (**D**) Representative fluorescent microscopy images of SKBR3 cells incubated with FITC-conjugated RrA in the absence or presence of Dynasore (green, FITC; blue, DAPI; magnification ×20). (**E**) Quantification of FITC-positive cells and (**F**) the FITC mean optical density (OD) in cells. # *p* ≤ 0.05 vs. cells incubated with RrA-FITC.

We investigated the influence of the inhibition of clathrin receptors on telomerase activity and hTERT expression by pre-treating Jurkat or SKBR3 cell lines with Dynasore and then incubating them with RrA. TRAP assay showed that clathrin receptor inhibition resulted in significant abolishment of telomerase inhibition by RrA in both cell lines (Figure 6A,B). Telomerase activity in Dynasore-exposed and RrA-treated cells was higher than in cells treated with RrA alone. Telomerase activity was associated with hTERT expression levels. HTERT mRNA levels in Dynasore-exposed and RrA-treated cells was higher than in cells treated with RrA alone (Figure 6C). Dynasore itself had no influence on telomerase activity or hTERT expression. These results indicate that clathrin receptor inhibition affects telomerase suppression by RrA.

Quantitative fluorescent microscopy demonstrated that the proportion of FITC-positive cells was significantly lower in Dynasore pre-treated cells (5.49% ± 2.01%) compared with RrA-FITC-incubated cells (68.21% ± 1.36%) (Figure 6D,E). The mean OD of FITC-positive cells was also decreased in Dynasore pre-treated cells (Figure 6F). The results obtained by flow cytometry are in good agreement with the flow cytometry results, which demonstrated the suppression of RrA-FITC interactions with cells in the presence of Dynasore (Table 3).

### 2.7. Chromatin Relaxation Leads to Complete Suppression of hTERT Expression and Telomerase Inhibition by RrA

**Figure 7:**
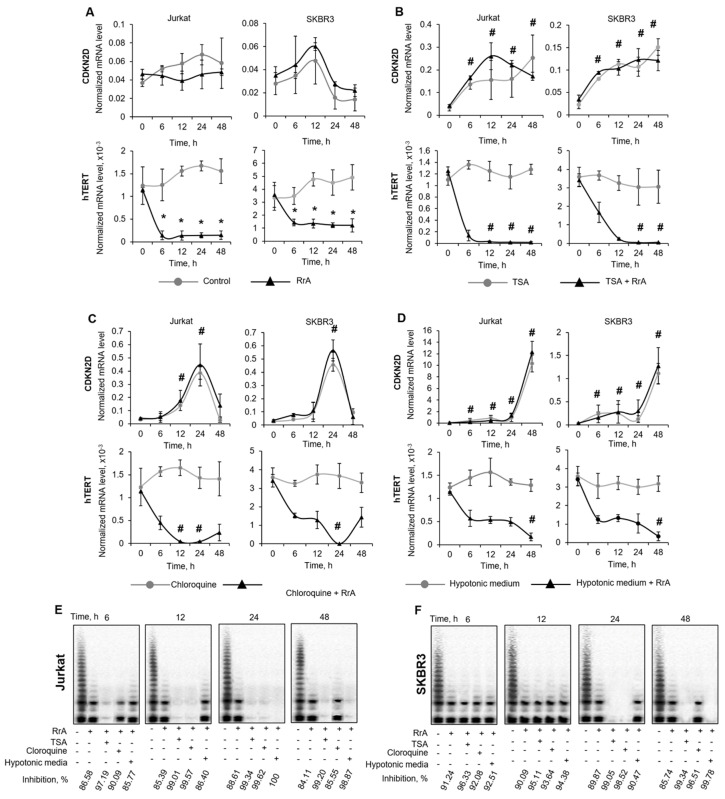
Chromatin relaxation increases the suppression rate of telomerase and hTERT expression by RrA. The Jurkat or SKBR3 cell lines were incubated under chromatin-modifying conditions in the presence of RrA. CDKN2D (marker of chromatin relaxation) and hTERT mRNA expression levels normalized relative to the expression of the reference gene 18S in (**A**) control Jurkat or SKBR3 cells; (**B**) cells treated with TSA; (**C**) cells treated with chloroquine or (**D**) cells grown in hypotonic medium. Representative TRAP gel electrophoresis and percent of inhibition of telomerase activity in (**E**) Jurkat or (**F**) SKBR3 cells. Percent of telomerase inhibition is calculated relative to untreated control cells. *N* = 4. RrA, *Rhodospirillum rubrum* L-asparaginase. TSA, trichostatin A. * *p* ≤ 0.05 vs. cells control untreated cells. # *p* ≤ 0.05 vs. cells incubated with RrA.

No single cell line showed close to 100% suppression of hTERT expression or telomerase inhibition in response to RrA treatment (Figure 2A,B, and Figure 7A). Chromatin conformation is known to impact the interaction efficiency of microbial proteins with eukaryotic DNA that is compacted in chromatin [14]. We investigated the influence of chromatin relaxation on the rate of telomerase suppression. We first analyzed whether an alteration in chromatin structure might trigger CDKN2D gene induction itself. To answer this question, we induced global chromatin relaxation in cells using the one of the following three chromatin-modifying agents: TSA, chloroquine, or hypotonic medium. All the treatments assayed caused a marked increase in CDKN2D expression in both cell lines. TSA was able to increase chromatin relaxation within 6–48 h in both cell lines (Figure 7B). Chloroquine was able to induce chromatin relaxation within 12–24 h (Figure 7C), whereas incubation of cells under hypotonic conditions led to most significant chromatin relaxation only at 48 h (Figure 7D). Chromatin relaxation resulted in increased inhibition of RrA activity. Complete suppression of hTERT expression along with telomerase activity was observed within 12–48 h in TSA-treated cells incubated with RrA (Figure 7B,E,F), within 12–24 h in chloroquine-treated cells incubated with RrA (Figure 7C,E,F) and at 48 h for cell grown under hypotonic conditions in the presence of RrA (Figure 7D–F).

## 3. Discussion

Most malignant tumors in humans have been demonstrated to depend on telomerase activity, and the catalytic subunit of the telomerase enzyme (i.e., hTERT) was found to be overexpressed in several tumors. Telomerase activity allows tumor cells to maintain the length of their telomere ends and escape replicative senescence; its regulatory role in metastatic events has also been proven. Many small molecule inhibitors of telomerase have been developed to date, however very few of them demonstrate significant antiproliferative or antitumor activity in vivo [15].

A very promising strategy for antitumor drug development is a development/synthesis of compounds that have dual activity and can inhibit more than one vital factor in cancer cells. In this regard, dual blockade of PD-L1 and MEK [16] or hybrid topoisomerase I and HDAC inhibitors have been developed as dual action anticancer agents [17]. Telomerase remains an attractive target for dual-blockade strategies, and several azidothymidines “clicked” into 1,2,3-triazoles were first reported to be carbonic anhydrase-telomerase dual-hybrid inhibitors [18].

An unexpected antitelomerase activity came from the observation that the rather well-studied L-ASNase from *Rhodospirillum rubrum* (RrA) can suppress proliferation and induces replicative senescence in acute leukemia Jurkat cancer cell line at non-toxic concentrations [11]. All known mechanisms of antiproliferative activity of L-ASNases rely on their ability to hydrolyze L-asparagine in the cell microenvironment or in a close proximity to cellular membranes. RrA appeared to be an enzyme that demonstrated dual activity and could suppress the expression of the telomerase main catalytic subunit hTERT, resulting in the inhibition of telomerase activity. In our work, we confirmed the ability of RrA among other L-ASNases to inhibit hTERT expression in different cancer cell lines (Figure 1 and Figure 2). Different cell lines showed different degrees of inhibition of telomerase and hTERT expression. In general, RrA could suppress hTERT expression up to 70–88%, which resulted in telomerase inhibition up to 27–91% in different cell lines. The reasons for this heterogeneity are highly speculative and remain to be investigated, though it is not surprising due to the rather complicated regulation of telomerase activity in diverse cells [19].

The mechanisms by which proteins penetrate eukaryotic cells are commonly studied using fluorescent labelling. Flow cytometry and fluorescence and confocal microscopy methods make it possible to estimate the amount of a fluorescently labeled protein that enters the cell and its distribution between cell compartments [20]. To answer the question about cellular localization of studied L-ASNases, we conjugated them to FITC and incubated these FITC-labeled enzymes with different cell lines. Only RrA demonstrated an ability to interact with cells, which was shown by flow cytometry and fluorescent microscopy (Figure 1D,E, Figure 2D,E, and Figure 3). We found rather a good connection between the rate of telomerase inhibition and the ability of RrA to interact with cells in different cell lines. RrA demonstrated the ability to penetrate cells, which corresponds to its capacity to inhibit hTERT expression. Other L-ASNases were not able to interact with cells (shown by flow cytometry) or penetrate into the cells. These results are also in accordance with a well-studied mechanism of their action based on asparagine deprivation.

To suppress hTERT expression, RrA must be localized in the cell nucleus. Confocal fluorescent microscopy revealed fluorescence signal in both the nucleus and nucleoli, that may suggest the nuclear and nucleolar localization of RrA-FITC (Figure 4). Further study is necessary to understand the mechanism by which RrA can inhibit hTERT, what other transcription factors are involved in this process and which other genes’ expression can be suppressed by RrA. Due to its low molecular mass (18 kDa), some amounts of RrA can enter the nucleus through passive transport, whereas accumulation inside the nucleolus may require a special NoLS protein signal sequence.

Unfortunately, a NoLS motif has not been detected in the RrA sequence. It has already been established that NLSs often function NoLSs and accumulate proteins inside the nucleolus [10]. We predicted two NLS motifs located at the N- and C-terminus of the RrA polypeptide chain. Therefore, we can only speculate that nucleus and nucleolar accumulation is through predicted NLS, which can also directly interact with nucleolar proteins [11].

The mechanism of RrA penetration through the cell membrane is of interest. Endocytosis is the process by which the cell absorbs dissolved compounds. Penetration by endocytosis involves the unspecific binding of cargo molecules to substances dissolved in the plasma membrane. Receptor-mediated endocytosis involves clathrin-dependent and -independent (caveolin-dependent) endocytosis. Receptor-mediated endocytosis provides highly selective absorption of molecules having a high affinity for receptors concentrated in regions where transport vesicles form [21]. We used different receptor-mediated endocytosis inhibitors to identify possible receptors involved in RrA penetration into cells (Figure 5A). Some amounts of RrA can pass through cell membranes by passive transport due to its small molecular size. However, sodium azide (NaN_3_) inhibited RrA penetration, which denotes the role of ATP-and GTP-dependent receptors (caveolin or clathrin) during this process. The inhibitor of clathrin receptors Dynasore was the most potent inhibitor of RrA penetration and could prevent the inhibition of hTERT expression and telomerase activity in Jurkat and SKBR3 cells (Figure 6). Dynamins represent a subfamily of GTP-binding proteins whose function is associated with clathrin receptors [22]. Quantitative real-time RT-PCR revealed good correspondence between the rates of hTERT suppression, telomerase inhibition by RrA and dynamin 2 (DNM2) mRNA levels in different cell lines (Figure 5B). Unfortunately, we did not find similar correspondence with the mRNA levels of the main protein of clathrin receptors (CLTC); however, our data showed possible involvement of clathrin receptors in the process of RrA translocation into cells.

Chromatin structure is closely related to many mechanisms involving DNA, such as replication, transcription, repair and recombination. Any event impairing chromatin stability is likely to compromise genome integrity and affect transcription. The most proven example is the activation of the expression of different deoxyribonucleases in response to the digestion of their promotors by the endonuclease EndoG [23]. Chromatin relaxation is used to increase the efficiency of gene editing [14]. The reason is that bacterial enzymes cannot sufficiently reach target regulatory sequences in condensed eukaryotic chromatin. DNA breaks or chromatin remodeling can relax chromatin and make target sites more abundant for prokaryotic proteins. We proposed that chromatin relaxation can increase the hTERT expression inhibition rate and consequently the rate of telomerase activity by the eukaryotic enzyme RrA. The above results suggest that chromatin structure alterations caused by chloroquine, TSA or hypotonic medium are sufficient to increase the rate of inhibition (Figure 7). This approach should be directly applicable when searching for other targets of RrA, considering its function as a transcriptional regulator.

## 4. Materials and Methods

### 4.1. L-Asparaginases and Conjugation with Fluorescein Isothiocyanate (FITC)

L-ASNases from *Rhodospirillum rubrum* (RrA), *Erwinia carotovora* (ErA), *Escherichia coli* (EcA), and *Wolinella succinogenes* (WsA) were used. The upstream, downstream, and enzymatic properties of the studied enzymes were described previously [24,25,26,27,28,29]. Conjugation of L-ASNases with FITC was carried out according to a described protocol [12]. Briefly, protein was dissolved in 0.1 M sodium carbonate buffer, pH 9, at concentration of 2 mg/mL and FITC (Sigma-Aldrich, St. Louis, MO, USA) in anhydrous DMSO was added to a final concentration of 50 µg/mL, followed by an 8 h incubation at 4 °C in the dark. Purification of the FITC-conjugated L-ASNases from unconjugated FITC was performed by gel-filtration using a Sephadex G25 PD-10 column (GE Healthcare, Chicago, IL, USA) in 0.1 M sodium carbonate buffer, pH 9. The conjugation efficiency was determined by the FITC/Protein (*F*/*P*) molar ratio. It is defined as the ratio of the moles of FITC to moles of protein with the conjugate and was calculated using the following equation:

(1) $$Molar\frac{F}{P}=\frac{MW}{389}*\frac{A_{495}/195}{\left[A_{280}-\left(0.35*A_{495}\right)\right]/E^{0.1\%}}=\frac{A_{495}*C}{A_{280}-[\left(0.35*A_{495}\right)]}\;\;\;$$

where, *MW* is the molecular weight of the protein; 389 is the molecular weight of FITC; 195 is the absorption E^0.1%^ of bound FITC at 490 nm and pH 13.0; A_280_ and A_495_ are the absorption of FITC-conjugated L-ASNase at given wavelengths; (0.35*A495) is the correction factor due to the absorbance of FITC at 280 nm; and E^0.1%^ is the absorption at 280 nm of a protein at concentration 1.0 mg/mL. An optimal conjugation efficiency has been considered to be in the range of 0.1–0.3 as recommended by the used protocol [12].

### 4.2. Cell Lines and Incubation with L-ASNases and the Inhibitors of Endocytosis

Acute T Cell Leukemia Jurkat cells, human cervical cancer HeLa cells, human chronic myelogenous leukemia K562 cells, human Burkitt’s Lymphoma Raji cells, human lung adenocarcinoma A549 cells, human breast cancer SKBR3 cells, human prostate cancer LnCap and PC-3 cells (all from ATCC, Manassas, VA, USA) were grown in RPMI-1640 (Thermo Fisher Scientific Inc., Waltham, MA, USA) supplemented with 5% fetal bovine serum (FBS, Capricorn Scientific, Ebsdorfergrund, Germany), cultivated in a 5% CO_2_/95% air humidified atmosphere at 37 °C. Adhesive cells were dissociated with 0.25% trypsin-EDTA (Thermo Fisher Scientific Inc., Waltham, MA, USA) and the medium was replaced with fresh complete medium every three-four days. Cells were incubated with L-ASNases or FITC-conjugated L-ASNases at a concentration 0.05 mg/mL for 3–12 h. The ability of FITC-conjugated L-ASNases to interact with cells was analyzed using flow cytometry (MACS Quant Analyzer 10, Miltenyi Biotec, Bergisch Gladbach, Germany) by counting FITC-positive cells and MFI was estimated for them.

To inhibit endocytosis, cells were pre-treated with an endocytosis receptor inhibitor for 30 min, followed by incubation with FITC-conjugated L-ASNases. The following inhibitors were used: the selective inhibitor of clathrin receptors chlorpromazine (230 µM, Sigma-Aldrich, St. Louis, MO, USA) [30]; an inhibitor of lipid-raft–mediated endocytosis pathways, including the caveolin-dependent endocytosis pathway, methyl-beta-cyclodextrin (MbCD, 10 µM, Alfa Aesar, Ward Hill, MT, USA) [31]; Dynasore (80 μM, Sigma-Aldrich, St. Louis, MO, USA), an inhibitor of dynamin-dependent endocytosis, in particular clathrin-dependent endocytosis [32]; or the non-selective universal inhibitor of ATP-dependent endocytosis sodium azide (NaN_3_, 100 mM, Paneco, Moscow, Russia) [21]. The inhibition efficiency of RrA-FITC interactions with cells (*U*) in the presence of the endocytosis inhibitors was calculated by the following equation:

(2) $$U=\;100-\frac{\left(X_{3}-X_{1}\right)}{\left(X_{2}-X_{1}\right)}\;\cdot 100\%\;,\mathrm{where}$$

*X*_1_ is the mean ratio of FITC-positive cells in control (untreated) cells;

*X*_2_ is the mean ratio of FITC-positive cells in the RrA-FITC-treated cells; and

*X*_3_ is the mean ratio of FITC-positive cells in cells treated with each of endocytosis inhibitor and RrA-FITC. The ability to interact with cells was considered as 100% for cells not exposed to any endocytosis inhibitors.

### 4.3. Chromatin Relaxation

Exponentially growing cells were incubated in fresh medium containing 200 nM trichostatin A (TSA) or 100 µM chloroquine (both from Sigma-Aldrich, St. Louis, MO, USA) for the indicated time intervals. For hypotonic treatment, cells were incubated in hypotonic medium (phosphate buffer saline, 0.45% glucose, 1% FSB, and 50 mM NaCl) for one hour [33]. Then, the hypotonic medium was replaced with fresh complete medium and the cells were incubated for the specific period of time indicated for each experiment. Cyclin dependent kinase inhibitor 2D (CDKN2D) mRNA expression levels were used as an indicator of chromatin relaxation [34].

### 4.4. Telomerase Activity Assay

Telomeric Repeat Amplification Protocol (TRAP) was perform to determine telomerase activity [35,36]. Cells were lysed in 10 mM Tris-HCl, pH 7.5, 1 mM MgCl_2,_ 1 mM EGTA, 0.1 mM PMSF, 5 mM 2-mercaptoethanol, 0.5% CHAPS and 10% glycerol (all from Sigma-Aldrich, St. Louis, MO, USA) and centrifuged for 30 min at 12000× *g*. The supernatants were stored at −80 °C. Elongation of the oligonucleotide substrate TS-primer (Telomerase Substrate primer) (5′-AATCCGTCGAGCAGAGTT-3′) and following amplification were conducted in a 30 μL reaction mixture containing 67 mM Tris-HCl, pH 8.8, 16.6 mM (NH_4_)_2_SO_4_, 0.01% Tween-20, 1.5 mM MgCl_2_, 1 mM EGTA (all from Sigma-Aldrich), 0.25 mM dNTPs (Syntol, Moscow, Russia) and 2 μL of cell lysate. Elongation was performed for 30 min at 37 °C followed by 10 min at 96 °C for telomerase inactivation. Then, 0.1 μL of CX-primer (Copy Extended primer) (5′-CCCTTACCCTTACCCTTACCCTAA-3′) and 2.5 units of Taq polymerase were added to the elongation mixture, followed by PCR using the following reaction conditions: (1) 94 °C for 5 min; (2) 30 cycles of 94 °C for 30 s, 50 °C for 30 s, and 72 °C for 40 s; and (3) 72 °C for 5 min. PCR product visualization was performed by 12% non-denaturing PAGE electrophoresis with TBE buffer. Ten microliters of samples were added to each well from a gel comb. The gels were stained with SYBR Green I (Invitrogen, Grand Island, NY, USA), photographed under UV light in a ChemiDoc™ XRS imaging system (Bio-Rad, Hercules, CA, USA) and analyzed with GelAnalyzer 2010a (Bio-Rad, Hercules, CA, USA).

### 4.5. RNA Isolation and Real-Time RT-PCR

PureLink RNA Mini Kit (Thermo Fisher Scientific Inc., Waltham, MA, USA) was used to extract total RNA from cells according to the manufacturer’s protocol. Reverse transcription and real-time RT-PCR were performed as previously described [37]. In total, 5 µg of total RNA was reverse-transcribed using an MMLV RT kit (Evrogen, Moscow, Russia) in a 25-µl reaction mixture, followed by real-time RT-PCR using DTprime5 (DNA Technology, Protvino, Russia). The reaction mix was prepared using qPCRmix-HS SYBR (Evrogen, Moscow, Russia) according to the manufacturer’s recommendations using the primers listed in Table S2 in the Supplementary file. Two annealing/extension temperature cycles were used. At the end of the annealing step measurement of the fluorescence was done. When the reaction reached its end, analysis of the Melting curve was performed (following the 45th cycle and between temperatures: 60 °C and 95 °C), to assess the final PCR product’s quality. For the reaction, effectiveness standard curves were displayed using 4 serially diluted samples (1:40, 1:80, 1:160, and 1:320) for hTERT or 18S cDNAs. hTERT mRNA levels were normalized relative to the expression of the reference gene 18S. The calculation of the relative RNA concentration was performed using the DTprime5 software.

### 4.6. Fluorescence Microscopy and Image Analysis

Cells were grown in 8 Chambers Cell Imaging Coverglass (Eppendorf, Hamburg, Germany), incubated with FITC-conjugated L-ASNases and fixed with 4% formalin. Control cells were treated with free FITC. Cells were then subjected to DNA counterstaining with 4′,6-diamidino-2-phenylindole (DAPI), mounted under a glass slide with the Prolong Antifade kit (Thermo Fisher Scientific Inc., Waltham, MA, USA) and imaged using a Leica DM4000 B LED (Leica Microsystems, Wetzlar, Germany) equipped with a Digital Color Camera Leica DFC310 FX (Leica Microsystems, Wetzlar, Germany).

Confocal microscopy was performed using a Nikon A1R MP+ laser scanning microscope (Nikon, Tokyo, Japan) with the Plan Apo 20×/0,75 Dic N or Apo IR 60×/1,27 WI objectives (Nikon, Tokyo, Japan). Lasers with wave lengths of 405 nm for DAPI and 488 nm for FITC were used.

Image analysis was performed using the NIS-Elements AR software (Nikon, Tokyo, Japan). For quantification, 10 independent fields of view were collected per each experimental subset, and the mean optical density (OD) was recorded for the channels used. Green and blue were used to mask FITC and nuclei stained with DAPI, respectively. Colocalization and pixel-to-pixel correlation analysis was performed using the Pearson and Manders algorithms available within the NIS-Elements AR software [38]. R coefficients for both correlations were calculated from 10 different images per experimental subset.

### 4.7. Prediction of Nuclear and Nucleolar Localization Signals

The presence of nuclear localization signal (NLS) motifs were predicted by the PSORT II [39], NucPred [40], NLStradamus [41], SeqNLS [42] or cNLSMapper [43] prediction programs from the amino acid sequences of the investigated L-ASNases. Evaluation of potential nucleolar localization signal (NoLS) was performed by the NOD program [44].

### 4.8. Statistics

Student’s t-test was performed using the Statistica 9.0 software v9.0 (StatSoft, Tulsa, OK, USA). Differences described as *p* ≤ 0.05 were considered significant. The results are presented as the mean±standard error of the mean (SEM) to indicate the uncertainty around the estimate of the mean measurement.

## 5. Conclusions

The results of this work show for the first time the intracellular localization of L-asparaginase from *Rhodospirillum rubrum* in human cancer cells. Clathrin-dependent endocytosis plays role in this process. This study demonstrated that different L-ASNases may have complex mechanisms of antineoplastic activity including regulation of transcription. Chromatin relaxation can increase the suppressive activity of RrA. Our findings show that RrA has the potential be used as an anticancer enzyme with a dual mechanism for antineoplastic activity.

## Figures and Tables

**Table 1:** F/P molar ratio values for the FITC-conjugated L-ASNases.

L-ASNase	MW	F/P Ration
RrA	18049.63	0.19
ErA	36551.55	0.14
WsA	36781.86	0.16
EcA	36850.38	0.19

ErA, *Erwinia carotovora* L-Asparaginase; EcA, *Escherichia coli* L-Asparaginase; F/P ratio, FITC, fluorescein isothiocyanate/protein ratio; L-ASNase, L-Asparaginase; MW; molecular weight; RrA, *Rhodospirillum rubrum* L-asparaginase; WsA, *Wolinella succinogenes* L-Asparaginase.

**Table 2:** Predicted NLS-like motifs in the RrA amino acid sequence.

Prediction Program	Predicted NLS	Score (Score Interval)
PSORT II	153-PAKTRKNR-160	−0.13
NucPred	No results	No results
NLStradamus	153-PAKTRKNRGLGR-164 156-TRKNR-160	0.1 (from 0.1 to 1.0) 0.2 (from 0.1 to 1.0)
SeqNLS	153-PAKTRKNR-160	0.433 (from 0.2 to 1.0)
cNLS Mapper	16-TIDKDYRLEENGLVVGDP FVAEVLKTARL-45	3 (from 2 to 7)
NOD	No results	No results

**Table 3:** The suppression efficiency (U, %) of RrA-FITC interactions with cells by the receptor-mediated endocytosis inhibitors.

Cell Line	Chlorpromazine	MbCD	Dynasore	NaN_3_
A549	12.3	20.9	75.2	76.1
K562	10.0	20.3	60.6	78.4
Jurkat	1.5	20.5	48.4	59.5
Raji	10.8	16.0	43.3	59.9
SKBR3	0.0	58.7	94.8	91.9
HeLa	10.4	18.3	69.4	83.1

MbCD, methyl-beta-cyclodextrin; NaN_3_, sodium azide.

## Supplementary Materials

The following are available online at https://www.mdpi.com/1424-8247/13/10/286/s1, Figure S1: The ability of endocytosis inhibitors at different concentrations to suppress RrA-FITC interactions with cells. Cells were pre-incubated for 30 min with endocytosis inhibitors at different concentrations and incubated with RrA-FITC for 3 h, followed by flow cytometry. Flow cytometry results for (A) Jurkat and (B) SKBR3 cells incubated with endocytosis inhibitors and RrA-FITC. N = 4. Representative flow cytometry diagrams for (C) Jurkat and (D) SKBR3 cells. Conc., concentration; MbCD, methyl-beta-cyclodextrin; NaN_3_, sodium azide; Figure S2: Representative flow cytometry diagrams demonstrating the ability of endocytosis inhibitors to suppress RrA-FITC interactions with cells. Cells were pre-incubated for 30 min with different endocytosis inhibitors and incubated with RrA-FITC for 3 h, followed by flow cytometry. RrA-FITC, *Rhodospirillum rubrum* L-asparaginase conjugated with fluorescein isothiocyanate, MbCD, methyl-beta-cyclodextrin; Figure S3: Representative flow cytometry diagrams demonstrating no cytotoxic effect of endocytosis inhibitors for cells. Cells were pre-treated with endocytosis receptor inhibitors for 30 min, followed by incubation with FITC-conjugated RrA. The following concentrations of the inhibitors were used: chlorpromazine, 230 µM; methyl-beta-cyclodextrin (MbCD), 10 µM; Dynasore, 80 μM; sodium azide (NaN_3_), 100 mM. Cells were incubated with 1µg/mL of propidium iodide followed by flow cytometry; Table S1: Amino acid sequences and molecular weights of investigated L-Asparaginases; Table S2: List of primers used for real-time RT-PCR.

## Abbreviations

AU	arbitrary units
ErA	*Erwinia carotovora* L-Asparaginase
EcA	*Escherichia coli* L-Asparaginase
DAPI	4′,6-diamidino-2-phenylindole
FITC	fluorescein isothiocyanate
hTERT	human telomerase reverse transcriptase
L-ASNase	L-Asparaginase
MbCD	methyl-beta-cyclodextrin
MFI	mean fluorescens intensity
NLS	nuclear localization signal
NoLS	nucleolar localization signal
OD	optical density
RrA	*Rhodospirillum rubrum* L-asparaginase
TSA	trichostatin A
TRAP	Telomeric Repeat Amplification Protocol
WsA	*Wolinella succinogenes* L-Asparaginase
