# Correction: Plyasova et al. Penetration into Cancer Cells via Clathrin-Dependent Mechanism Allows L-Asparaginase from *Rhodospirillum rubrum* to Inhibit Telomerase. *Pharmaceuticals* 2020, *13*, 286

**DOI:** 10.3390/ph17060684

**Published:** 2024-05-27

**Authors:** Anna A. Plyasova, Marina V. Pokrovskaya, Olga M. Lisitsyna, Vadim S. Pokrovsky, Svetlana S. Alexandrova, Abdullah Hilal, Nikolay N. Sokolov, Dmitry D. Zhdanov

**Affiliations:** 1 Institute of Biomedical Chemistry, Pogodinskaya st. 10/8, 119121 Moscow, Russia; annaplyasova13@gmail.com (A.A.P.); ivan1190@ya.ru (M.V.P.); mbt12@yandex.ru (S.S.A.); abdulla.hilal@inbox.ru (A.H.); nikolai.sokolov@ibmc.msk.ru (N.N.S.); 2 International Biotechnology Center “Generium” LLC, Vladimirskaya st. 14, 601125 Volginsky, Russia; lisitsynaom@gmail.com; 3 N.N. Blokhin Cancer Research Center, Kashirskoe Shosse 24, 115478 Moscow, Russia; v.pokrovsky@ronc.ru; 4 Department of Biochemistry, Peoples Friendship University of Russia (RUDN University), Miklukho-Maklaya st. 6, 117198 Moscow, Russia

In the original publication [1], there was a mistake in Figure 3 as published. This accident occurred as a result of working with many captures. The corrected Figure 3 appears below. The authors state that the scientific conclusions are unaffected. This correction was approved by the Academic Editor. The original publication has also been updated.

## Figures and Tables

**Figure 3:**
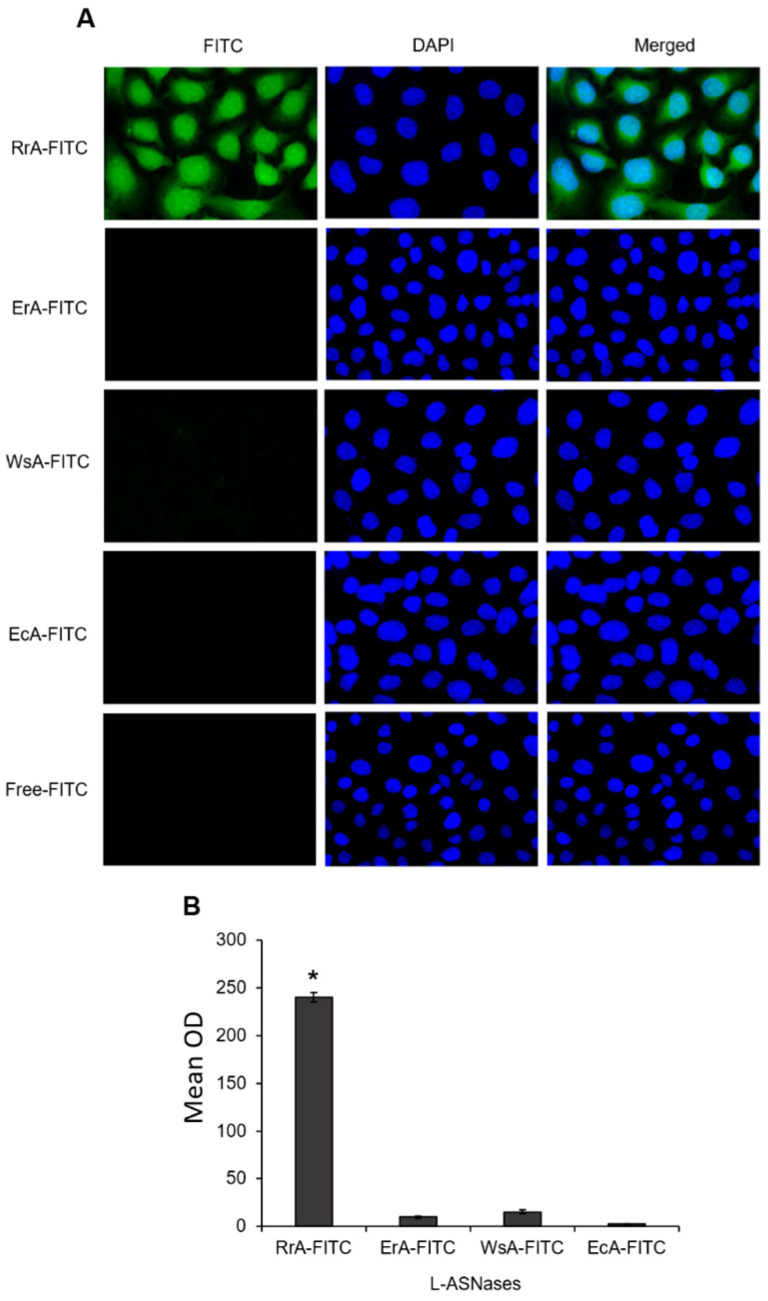
Induction of FITC-positive cells after treatment with FITC-conjugated L-ASNases. (**A**) Representative fluorescent microscopy images of SKBR3 cell incubated with different FITC-conjugated L-ASNases for 12 h and stained with DAPI (green, FITC, and blue, DAPI, magnification ×40). (**B**) Quantification of the FITC mean optical density (OD) in cells. * *p* ≤ 0.05 vs. cells incubated with free FITC.
